# Mitosis-related phosphorylation of the eukaryotic translation suppressor 4E-BP1 and its interaction with eukaryotic translation initiation factor 4E (eIF4E)

**DOI:** 10.1074/jbc.RA119.008512

**Published:** 2019-06-14

**Authors:** Rui Sun, Erdong Cheng, Celestino Velásquez, Yuan Chang, Patrick S. Moore

**Affiliations:** ‡ Department of Microbiology and Molecular Genetics, University of Pittsburgh, Pittsburgh, Pennsylvania 15213; ¶ Department of Pathology, University of Pittsburgh, Pittsburgh, Pennsylvania 15213; § Cancer Virology Program, UPMC Hillman Cancer Center, Pittsburgh, Pennsylvania 15213

**Keywords:** eukaryotic translation initiation factor 4E (eIF4E), eukaryotic translation initiation factor 4E-binding protein 1 (EIF4EBP1), eukaryotic translation initiation factor 4G (eIF4G), eukaryotic translation initiation, cell cycle, mitosis, protein phosphorylation, mammalian target of rapamycin (mTOR), cyclin-dependent kinase 1 (CDK1), PHAS-I

## Abstract

Eukaryotic translation initiation factor 4E (eIF4E)–binding protein 1 (4E-BP1) inhibits cap-dependent translation in eukaryotes by competing with eIF4G for an interaction with eIF4E. Phosphorylation at Ser-83 of 4E-BP1 occurs during mitosis through the activity of cyclin-dependent kinase 1 (CDK1)/cyclin B rather than through canonical mTOR kinase activity. Here, we investigated the interaction of eIF4E with 4E-BP1 or eIF4G during interphase and mitosis. We observed that 4E-BP1 and eIF4G bind eIF4E at similar levels during interphase and mitosis. The most highly phosphorylated mitotic 4E-BP1 isoform (δ) did not interact with eIF4E, whereas a distinct 4E-BP1 phospho-isoform, EB-γ, phosphorylated at Thr-70, Ser-83, and Ser-101, bound to eIF4E during mitosis. Two-dimensional gel electrophoretic analysis corroborated the identity of the phosphorylation marks on the eIF4E-bound 4E-BP1 isoforms and uncovered a population of phosphorylated 4E-BP1 molecules lacking Thr-37/Thr-46–priming phosphorylation. Moreover, proximity ligation assays for phospho-4E-BP1 and eIF4E revealed different *in situ* interactions during interphase and mitosis. The eIF4E:eIF4G interaction was not inhibited but rather increased in mitotic cells, consistent with active translation initiation during mitosis. Phosphodefective substitution of 4E-BP1 at Ser-83 did not change global translation or individual mRNA translation profiles as measured by single-cell nascent protein synthesis and eIF4G RNA immunoprecipitation sequencing. Mitotic 5′-terminal oligopyrimidine RNA translation was active and, unlike interphase translation, resistant to mTOR inhibition. Our findings reveal the phosphorylation profiles of 4E-BP1 isoforms and their interactions with eIF4E throughout the cell cycle and indicate that 4E-BP1 does not specifically inhibit translation initiation during mitosis.

## Introduction

4E-BP1,^5^ also known as phosphorylated heat- and acid-stable protein regulated by insulin (PHAS-I), was first identified as a protein phosphorylated in response to insulin treatment (1). 4E-BP1 was subsequently isolated from a human cDNA library of eIF4E-binding proteins and shown to inhibit cap-dependent translation (2, 3). Efficient cap-dependent translation requires assembly of the translation initiation complex eIF4F (composed of eIF4E, eIF4G, and eIF4A) on the mRNA 5′ cap structure (4, 5). 4E-BP1 inhibits translation by binding to eIF4E, which prevents eIF4G:eIF4E interaction, thus inhibiting assembly of the eIF4F complex (6–14).

4E-BP1 is a small 15-kDa protein (118 amino acids in humans and 117 amino acids in rodents). At least seven human 4E-BP1 phosphorylation sites have been identified and validated, which include Thr-37, Thr-46, Ser-65, Thr-70, Ser-83, Ser-101, and Ser-112 (15–18). When 4E-BP1 is hyperphosphorylated (p-4E-BP1^T37/T46,^ ^S65, T70^), it no longer sequesters eIF4E, allowing eIF4G:eIF4E interaction and initiation of cap-dependent translation (19–22). Mechanistic target of rapamycin complex 1 (mTORC1) is the primary kinase controlling 4E-BP1–regulated translation during interphase (23–25). When mTORC1 is inhibited, 4E-BP1 becomes dephosphorylated, which increases 4E-BP1 affinity for eIF4E. Data suggest that this preferentially inhibits the translation of a subset of capped mRNAs containing 5′-terminal oligopyrimidine (TOP) tracts (26, 27); however, some data do not (28). mTORC1-mediated phosphorylation of 4E-BP1 has been recognized as a critical control point for many cancers, leading to the application of mTOR inhibitors in cancer chemotherapies (29).

Several conserved motifs have been identified in the protein structure of 4E-BP1 (30). Motif 1 (^54^Y*XXXX*LΦ^60^) is responsible for direct eIF4E binding (6, 7, 14). The priming phosphorylation sites Thr-37/Thr-46 adjacent to motif 1 are targeted by mTORC1 (23–25). Motif 2 is a proline–turn–helix segment containing phosphorylation sites Ser-65 and Thr-70. It has been suggested, in a two-step model, that priming phosphorylation at Thr-37/Thr-46 is required for subsequent phosphorylations at Ser-65 and Thr-70, which then render hyperphosphorylated 4E-BP1 unable to bind eIF4E (19, 20). Motif 3 (^70^IPGVTSP^84^) is a C-terminal loop of 4E-BP1 required for high-affinity association with eIF4E (8, 9, 11–13). Furthermore, 4E-BP1 has an N-terminal RAIP motif and a C-terminal TOS motif, which also take part in regulating its phosphorylation (31–33).

In contrast to 4E-BP1 residues Thr-37, Thr-46, Ser-65, and Thr-70, which are phosphorylated during interphase, 4E-BP1 Ser-83 is phosphorylated only during mitosis by the cyclin-dependent kinase 1 (CDK1)/cyclin B complex, providing a unique marker for mitosis (34). CDK1/cyclin B can also substitute for mTOR during mitosis to phosphorylate other sites, including Thr-37/Thr-46 (35). Thus, 4E-BP1 exhibits different phosphorylation patterns throughout the cell cycle. However, it remains unknown whether this phenomenon results in different eIF4E:4E-BP1 interactions.

Here, we examined 4E-BP1 phosphorylation and 4E-BP1:eIF4E interaction throughout the cell cycle in HeLa cells. A distinct eIF4E-binding (EB)-γ isoform of 4E-BP1, with a phosphorylated Ser-83 residue, was identified to bind eIF4E during mitosis, demonstrating that Ser-83 phosphorylation alone does not prevent 4E-BP1 from sequestering eIF4E. The combinatorial complexity of the various phosphorylation sites on 4E-BP1 have largely, and unsatisfactorily, been resolved using various phosphospecific antibodies via one-dimensional gel electrophoresis. At best, four closely migrating protein bands designated α, β, γ, and δ are distinguishable. By differentiating 4E-BP1 isoforms on two-dimensional gel electrophoresis, multiple new phospho-isoforms of 4E-BP1 were identified, including phospho-isoforms lacking priming phosphorylations at Thr-37/Thr-46. Concurrently, we characterized the key 4E-BP1 phosphorylation events for the regulation of the 4E-BP1:eIF4E interaction, expanding the previously proposed two-step model. Proximity ligation assays (PLAs) provided visual localization of the *in situ* interaction between eIF4E and different phosphorylated 4E-BP1 isoforms during mitosis and interphase. Strong eIF4E:eIF4G PLA signals were present in mitotic cells, suggesting that assembly of the translation initiation eIF4F complex is not inhibited but rather increased in mitosis. In contrast to previously examined cell lines (35), 4E-BP1–independent global translation suppression was observed in HeLa cells by a flow cytometry–based Click-iT labeling assay, which indicates that mitotic translation inhibition occurs downstream of eIF4F complex loading to RNA. eIF4G RNA immunoprecipitation sequencing (RIP-Seq) validated active mitotic TOP gene translation initiation, consistent with 4E-BP1 not being responsible for mitotic translation suppression in HeLa cells. Alanine substitution mutation at 4E-BP1^S83^ alone did not significantly alter eIF4G RIP-Seq profiles. Taken together, these data reveal phosphorylation marks on eIF4E-associated 4E-BP1 isoforms throughout the cell cycle and update the understanding of various 4E-BP1 phosphorylation marks on 4E-BP1 function.

## Results

### Cell cycle–related phospho-4E-BP1 binding to eIF4E

**Figure 1.**
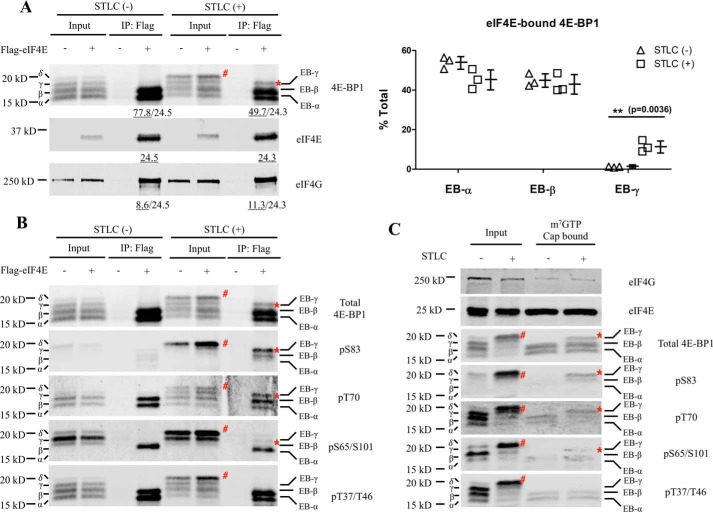
**Cell cycle–dependent differences in phospho-4E-BP1 binding to eIF4E.** *A*, FLAG-tagged eIF4E plasmids were transfected into HeLa cells. Transfected cells were split into two groups, 1) asynchronous and 2) synchronized at mitosis, by STLC treatment (5 μm; 16 h). Cell lysates were immunoprecipitated with anti-FLAG antibodies followed by immunoblotting with corresponding antibodies. The intensities of immunoprecipitated bands were quantitated (underlined values). The ratio of each eIF4E-bound 4E-BP1 band in total was calculated (*right panel*). Results are presented as mean ± S.D. *Error bars* represent S.D. The *p* value was calculated by *t* test with **, *p* < 0.01. At least three biological replicates were performed. Data shown here is a representative result. The immunoprecipitated 4E-BP1 and eIF4G levels are normalized to immunoprecipitated eIF4E band intensities. *B*, the membrane from *A* was stripped and reprobed with different phosphospecific 4E-BP1 antibodies. Total 4E-BP1 immunoblotting from *A* is shown for comparison. *C*, HeLa cells were split into asynchronous cells and STLC-treated (5 μm; 16 h) mitosis-enriched cells. Cell lysates were incubated with m^7^GTP cap pulldown beads. Cap-bound proteins were detected by immunoblotting with the designated antibodies. The 4E-BP1 EB-γ isoform is indicated by *, and the 4E-BP1 δ isoform is indicated by #. EB-γ and γ are two different and distinct 4E-BP1 phospho-isoforms.

SDS-PAGE immunoblotting revealed α, β, γ, and δ 4E-BP1 phospho-isoforms (Fig. 1*A*) (35) with the highest molecular mass (slowest migrating) isoform (∼20 kDa), designated the δ band, enriched in mitosis-arrested cells after *S*-trityl-l-cysteine (STLC) treatment (34, 35). eIF4E pulldown showed similar or modestly decreased levels of 4E-BP1 binding to eIF4E during mitosis as compared with interphase. No eIF4E interaction was detected with the most highly phosphorylated δ 4E-BP1 isoform. However, three phosphorylated, lower-molecular-weight 4E-BP1 bands (designated EB-α, -β, and -γ), coimmunoprecipitated with eIF4E. Of these three bands, the less abundant, but slowest migrating 4E-BP1 band (EB-γ) was enriched in mitosis-arrested cell extracts. Similar or slightly increased amounts of eIF4G coimmunoprecipitated with eIF4E from mitosis-arrested cells when compared with asynchronous cells, suggesting that assembly of the translation initiation complex eIF4F was not specifically inhibited in mitosis.

To determine the phosphorylation profiles of eIF4E-bound 4E-BP1 isoforms, the membrane was stripped and reprobed with phosphospecific 4E-BP1 antibodies (Fig. 1*B*). The eIF4E-unbound δ band was positive for Ser-83, Thr-37/Thr-46, Ser-65/Ser-101, and Thr-70 phosphorylations. The eIF4E-immunoprecipitated EB-α and EB-β bands present in both mitotic and asynchronous cells were positive for Thr-37/Thr-46 and Thr-70 phosphorylations, suggesting that phosphorylation at the Thr-37/Thr-46 priming sites and/or Thr-70 is insufficient to dissociate 4E-BP1 from eIF4E (19, 20). The mitotic EB-γ band was positive for Ser-83 and Thr-70 phosphorylations but not for priming phosphorylations at Thr-37/Thr-46. To rule out artifacts due to eIF4E overexpression, the interaction of endogenous eIF4E with 4E-BP1 was determined by m^7^GTP cap pulldown assays as well and showed similar results (Fig. 1*C*).

**Figure 2.**
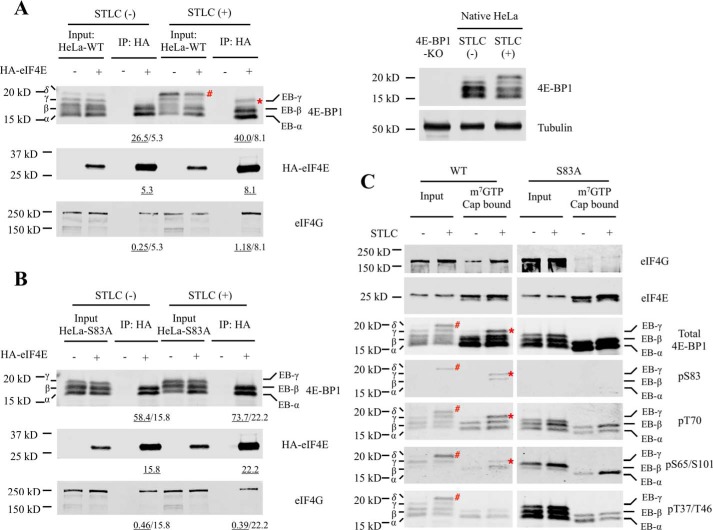
**Phosphodefective substitution of 4E-BP1 at Ser-83 eliminates the EB-γ isoform.** WT 4E-BP1 (*A*) or 4E-BP1^S83A^ mutant (*B*) was stably expressed in HeLa-4E-BP1–knockout (*KO*) cells. No endogenous 4E-BP1 is present in HeLa-4E-BP1–knockout cells (*right*). The eIF4E-transfected WT 4E-BP1 or 4E-BP1^S83A^ mutant cells were then divided into asynchronous (*Async.*) cells and STLC-treated (5 μm; 16 h) mitosis-enriched cells. Cell lysates were immunoprecipitated with anti-HA antibodies followed by immunoblotting with the designated antibodies. The intensity of eIF4E-bound band was quantitated and annotated (underlined values). The immunoprecipitated 4E-BP1 and eIF4G level are normalized by immunoprecipitated eIF4E band intensity. *C*, WT 4E-BP1 or 4E-BP1^S83A^ mutant was stably expressed in HeLa-4E-BP1–knockout cells. Cells were then divided into asynchronous cells and STLC-treated (5 μm; 16 h) mitosis-enriched cells. Cell lysates were incubated with m^7^GTP cap pulldown beads. Cap-bound proteins were detected by immunoblotting with the designated antibodies. 4E-BP1 EB-γ isoform is indicated by *, and 4E-BP1 δ isoform is indicated by #.

To confirm the presence of Ser-83 phosphorylation in the EB-γ isoform of 4E-BP1, a 4E-BP1–knockout HeLa cell line was generated by CRISPR/Cas9. WT 4E-BP1 or various 4E-BP1 mutants were stably expressed in the 4E-BP1–knockout HeLa cells. Alanine substitution mutation at 4E-BP1 Ser-83 (4E-BP1^S83A^) eliminated the δ isoform from mitosis-arrested cells (Fig. 2, *A* and *B*). Similarly, the EB-γ isoform, which was detected in the mitotic WT 4E-BP1 cells, was absent from mitotic 4E-BP1^S83A^ mutant cells (Fig. 2, *A* and *B*). This was further confirmed by m^7^GTP cap pulldown assays (Fig. 2*C*). The EB-γ isoform of 4E-BP1, phosphorylated at Ser-83, Thr-70, and Ser-65/Ser-101, retained interaction with the m^7^GTP cap (Fig. 2*C*), indicating that mitotic Ser-83 phosphorylation alone is not sufficient to block 4E-BP1 sequestration of eIF4E.

### Phospho-4E-BP1 isoforms identified in mitosis

**Figure 3.**
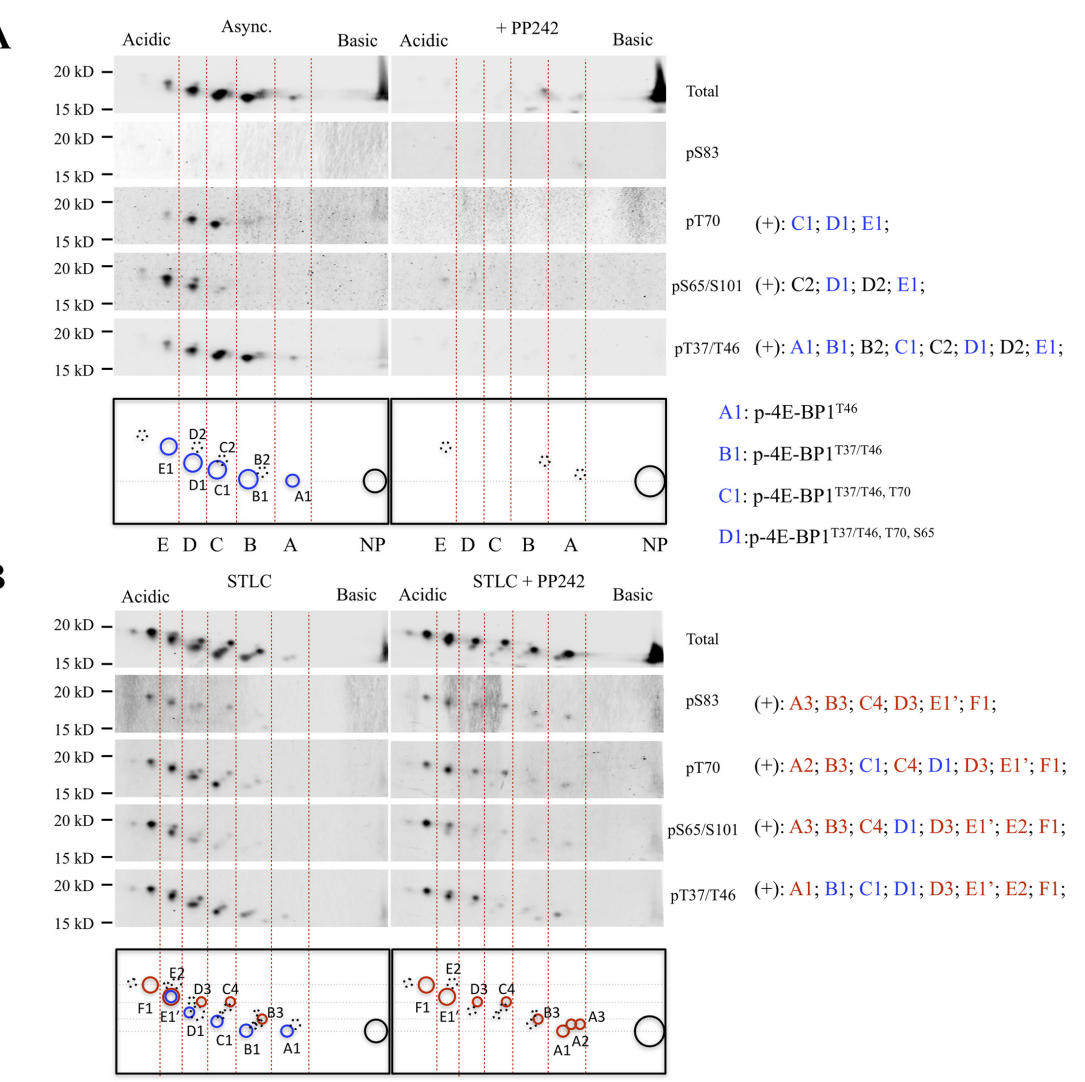
**Phospho-4E-BP1 isoforms identified in mitosis.** Cell lysates collected from asynchronous and mTOR inhibitor PP242-treated (5 μm; 4 h) HEK 293 cells (*A*) or STLC-arrested (5 μm; 16 h) HEK 293 cells treated with or without mTOR inhibitor PP242 (5 μm; 4 h) (*B*) were subjected to 2D-gel electrophoresis (isoelectric focusing at pH 3–6) followed by immunoblotting with different phosphospecific and total 4E-BP1 antibodies. *Blue circles* indicate canonical phospho-isoforms (20, 37), *red circles* indicate PP242-resistant isoforms of 4E-BP1 in mitosis, *dashed-line circles* indicate additional isoforms with weaker signals, and *NP* indicates nonphosphorylated 4E-BP1.

Two-dimensional gel (2D-gel) electrophoresis separated phosphorylated 4E-BP1 isoforms into five isoelectric groups (A–E) for asynchronous cells (Fig. 3*A*) and six isoelectric groups (A–F) for mitosis-arrested cells (Fig. 3*B*). Within each isoelectric group (*e.g.* A), each subnumber (*e.g.* A1) represents a distinguishable charge–mass isoform. Phosphoreactivity of each major dot is shown in the *right panels* in Fig. 3.

For asynchronous cells, mTOR inhibitor PP242 treatment ablated all detectable 4E-BP1 phosphorylation (Fig. 3*A*), consistent with previously published results (19, 36). For mitosis-arrested cells, 4E-BP1 phosphorylations at multiple residues (Thr-37/Thr-46, Ser-65/Ser-101, Thr-70, and Ser-83) were resistant to PP242 treatment (Fig. 3*B*), confirming mTOR-independent phosphorylation during mitosis (34). Mitosis-arrested cells showed high levels of hyperphosphorylated 4E-BP1 (E and F) compared with asynchronous cells. Notably, Ser-83 phosphorylation was only detectable in mitosis-arrested cells. Lower-order isoforms (Fig. 3*B*, dots A3 and B3) with Ser-83 phosphorylation appeared upon PP242 treatment, suggesting that mTOR-dependent phosphorylation at sites other than Ser-83 also contributed to the mitotic hyperphosphorylated 4E-BP1 population.

The lowest-order 4E-BP1 phospho-isoform in asynchronous cells (Fig. 3*A*, *dot A1*) showed PP242-sensitive Thr-37/Thr-46 phosphorylation. This phosphorylation was present in most higher-order phospho-isoforms (B, C, D, and E), consistent with mTOR-dependent Thr-37/Thr-46–priming phosphorylation during interphase as reported previously (20). For mitotic cells, the most highly phosphorylated isoforms (F), corresponding to the δ band seen on 1D gel, were abundant and resistant to PP242 treatment (Fig. 3*B*). Multiple mitotic lower-order phospho-isoforms lacked Thr-37/Thr-46–priming phosphorylations and were also resistant to PP242 treatment (*e.g.* dots A2, A3, B3, and C4). Based on its migration and phosphorylated residues, dot C4 most likely represents the EB-γ band found in Fig. 1. This was confirmed by alanine substitution mutation at 4E-BP1 Ser-83, which eliminated the isoforms containing Ser-83 phosphorylation (*e.g.* dots C4 and F) (Fig. S1). The mitotic 4E-BP1 phosphorylation pattern determined in STLC-treated cells was also validated with mitotic cells collected by the mitotic shake-off method (Fig. S2).

### Two-dimensional profile of eIF4E-bound 4E-BP1 isoforms

**Figure 4.**
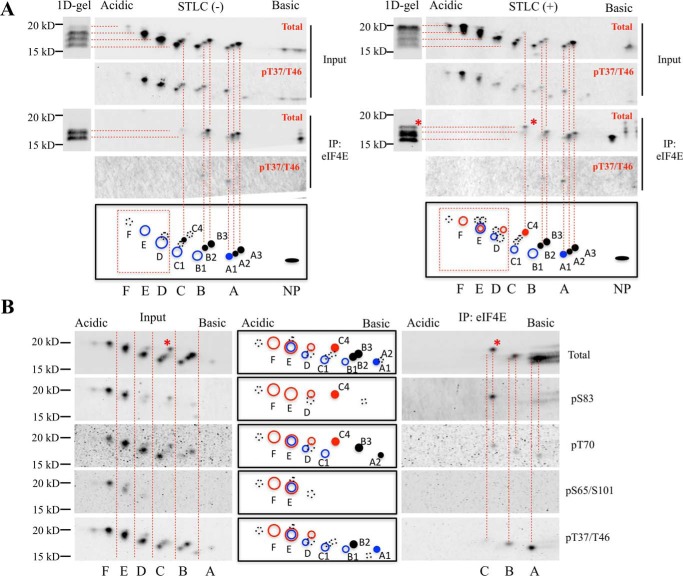
**Two-dimensional profile of eIF4E-bound 4E-BP1 isoforms.** *A*, HA-tagged eIF4E expression plasmids were transfected into HEK 293 cells. Transfected cells were divided into two groups, 1) asynchronous and 2) synchronized at mitosis, by STLC treatment (5 μm; 16 h). Cell lysates were immunoprecipitated for eIF4E with anti-HA antibodies. Cell lysates (*Input*) or immunoprecipitated elutes (*IP*) were subjected to 1D- and 2D-gel electrophoresis (isoelectric focusing at pH 3–6) followed by immunoblotting with total 4E-BP1 and p-4E-BP1^T37/T46^ antibodies. *B*, FLAG-tagged eIF4E plasmids were transfected into HeLa cells. Transfected cells were synchronized at mitosis with STLC (5 μm; 16 h). Cell lysates were immunoprecipitated with anti-FLAG antibodies. Cell lysates (*Input*) or immunoprecipitated elutes (*IP*) were subjected to 2D-gel electrophoresis (isoelectric focusing at pH 3–6) followed by immunoblotting with different phosphospecific and total 4E-BP1 antibodies. *Blue circles* indicate canonical phosphorylated 4E-BP1 isoforms (20, 37), *red circles* indicate PP242-resistant isoforms of 4E-BP1 in mitosis, *dashed-line circles* indicate isoforms with weaker signals, *filled circles* indicate eIF4E-bound 4E-BP1 isoforms, and *NP* indicates nonphosphorylated 4E-BP1. The 4E-BP1 EB-γ isoform is indicated by *.

To determine the phosphorylation profile of the eIF4E-bound 4E-BP1 isoforms on 2D gels, 2D-gel electrophoresis was performed after eIF4E coimmunoprecipitation (Fig. 4*A*). Hyperphosphorylated 4E-BP1 (D, E, and F) showed no interaction with eIF4E, whereas three lower-order 4E-BP1 phospho-isoforms (A, B, and C) bound to eIF4E in both asynchronous and mitosis-arrested cells, consistent with hypophosphorylated 4E-BP1 sequestering eIF4E. A greater fraction of mitotic 4E-BP1 was hyperphosphorylated (E and F) compared with asynchronous cells. Comparison of input lysate with eIF4E-immunoprecipitated 4E-BP1 revealed reduced A1 and B2 immunoprecipitation compared with isoforms A2, A3, B3, and C4, suggesting that phosphorylation at the Thr-37/Thr-46 priming sites alone substantially weakens eIF4E:4E-BP1 interaction but is still not sufficient to block 4E-BP1 sequestration of eIF4E. The dot at position C4 aligns with the EB-γ band identified on 1D-gel electrophoresis (Fig. 1) and was enriched in mitotic cells. The eIF4E-immunoprecipitated dot C4 showed phosphorylation at both Ser-83 and Thr-70 but was negative for Thr-37/Thr-46–priming phosphorylation as shown in Fig. 4*B*. Most of the eIF4E-bound isoforms of 4E-BP1 during mitosis were newly identified phosphorylated isoforms: dots A2, A3, B3, and C4. The previously presumed hypophosphorylated isoforms (dots B1 and C1) (19) had no interaction with eIF4E, suggesting that phosphorylation events occurring at dot B1 are the key control point for eIF4E:4E-BP1 interaction. Dot B1 is positive for Thr-37/Thr-46 phosphorylation and negative for other known phosphorylations, which is consistent with previous studies (20, 37). It most likely represents an isoform of 4E-BP1 phosphorylated at both Thr-37 and Thr-46 because alanine substitution mutation at Thr-37 or Thr-46 eliminated the B1 isoform (Fig. S3).

### Mitotic 4E-BP1:eIF4E and eIF4G:eIF4E in vivo interactions

**Figure 5.**
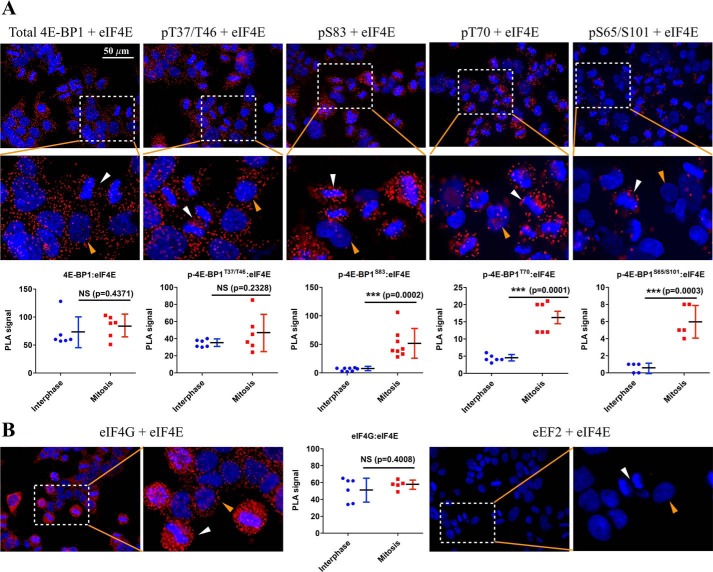
**Mitotic 4E-BP1:eIF4E and eIF4G:eIF4E interactions *in vivo*.** HeLa cells were synchronized at the G_2_/M boundary with CDK1 inhibitor RO3306 treatment (10 μm; 16 h) and then released into mitosis by removing RO3306. After 60 min, cells were fixed and permeabilized. *A*, PLAs were performed using mouse eIF4E and rabbit phosphospecific or total 4E-BP1 antibodies. Cell nuclei were stained with DAPI (*blue*). PLA signal was obtained from rolling circle amplification (*red*). Images were captured by fluorescence microscope (40×). *B*, PLAs were performed using mouse eIF4E and rabbit eIF4G or eEF2 antibodies. Images were captured by fluorescence microscope (40×). *White arrows* indicate mitotic cells; *yellow arrows* indicate interphase cells. PLA signals were quantitated using ImageJ (particle counting). Results are presented as mean ± S.D. *Error bars* represent S.D. The *p* value was calculated by *t* test. *NS* indicates that the difference is not significant. ***, *p* < 0.001.

To investigate mitotic 4E-BP1:eIF4E and eIF4G:eIF4E interaction *in vivo*, PLAs were used to detect *in situ* eIF4E interactions in HeLa cells (Fig. 5). Positive PLA signals between eIF4E and total 4E-BP1, p-4E-BP1^T37/T46^, p-4E-BP1^S83^, p-4E-BP1^T70^, or p-4E-BP1^S65/S101^ were all detected, but the pattern and amount of positive fluorescence dots varied among different 4E-BP1 phosphorylations (Fig. 5*A*). Phospho-4E-BP1^S83^ PLA interactions with eIF4E were restricted to mitotic cells, whereas p-4E-BP1^T37/T46^ and p-4E-BP1^T70^ interactions with eIF4E were present in both mitotic and interphase cells. PLA interaction between eIF4E and p-4E-BP1^S65/S101^ was almost undetectable in interphase cells and weakly increased in mitotic cells. Phospho-4E-BP1^S83^ and eIF4E diffusely colocalized during mitosis, consistent with a portion of p-4E-BP1^S83^ retaining eIF4E sequestration activity (Figs. 1, 2, and 4). Strong fluorescent signals were observed across all stages of mitosis (Fig. S4).

The dephosphorylation of 4E-BP1 has been proposed to be responsible for the shutdown of mitotic cap-dependent translation (38). This has been disputed in several recent studies showing high levels of 4E-BP1 phosphorylation (34, 35, 39, 40) and active cap-dependent translation during mitosis using single-cell pulse-chase analysis (35). Even though a substantial fraction of eIF4E was found to be bound to 4E-BP1 during both mitosis and interphase (Fig. 5*A*), strong fluorescent eIF4E:eIF4G PLA signals were present in mitotic cells, suggesting that assembly of the translation initiation eIF4F complex is not inhibited, confirming the eIF4E coimmunoprecipitation (co-IP) results (Figs. 5*B*, 1*A*, and 2, *A* and *B*) as well as previously published studies (35, 41).

### Global mitotic translation in HeLa cells

**Figure 6.**
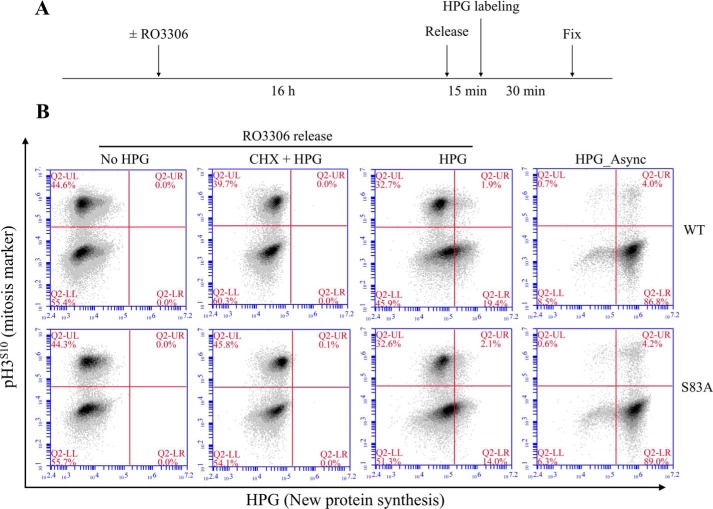
**Phosphodefective substitution of 4E-BP1 at Ser-83 does not change global translation.** *A*, illustration of the mitotic Click-iT labeling assay. WT 4E-BP1 or 4E-BP1^S83A^ mutant HeLa cells were synchronized at the G_2_/M boundary with CDK1 inhibitor RO3306 treatment (10 μm; 16 h) and then released into mitosis by removing RO3306. After incubating with methionine-depleted medium for 15 min, cells were treated with HPG (50 μm) for 30 min. Cycloheximide (*CHX*; 100 μg/ml) was added at the same time to block new protein synthesis, functioning as the negative control. Cells were collected and fixed for subsequent staining. *B*, flow cytometry analysis of HPG incorporation (new protein synthesis). Cells were labeled with Alexa Fluor 488 azide using Click-iT HPG kits and stained with p-H3^S10^ antibody to label the mitotic cell population.

To determine whether Ser-83 phosphorylation of 4E-BP1 affects global translation, single-cell protein synthesis was measured in WT 4E-BP1 and 4E-BP1^S83A^ mutant HeLa cells by a flow cytometry–based Click-iT labeling assay (35). Newly synthesized proteins are labeled by the methionine analog l-homopropargylglycine (HPG) in a pulse-chase assay. To specifically label mitotic, newly synthesized proteins, cells were arrested at the G_2_/M boundary with the CDK1 inhibitor RO3306, and HPG was added to the methionine-depleted medium following RO3306 release. As shown in Fig. 6, repressed translation was observed in a large population of mitotic cells (p-H3^S10^–positive), consistent with previously reported translation assay results for HeLa cells (42, 43) but different from the observed results in BJ-T cells (35). This repression was not due to 4E-BP1 dephosphorylation as the same repression was also observed in 4E-BP1–knockout and native HeLa cells (Fig. S5). No significant differences were found between WT 4E-BP1 and 4E-BP1^S83A^ mutant HeLa cells, suggesting that Ser-83 phosphorylation of 4E-BP1 does not affect global translation in HeLa cells.

### Mitotic 5′-TOP transcript translation in HeLa cells

**Figure 7.**
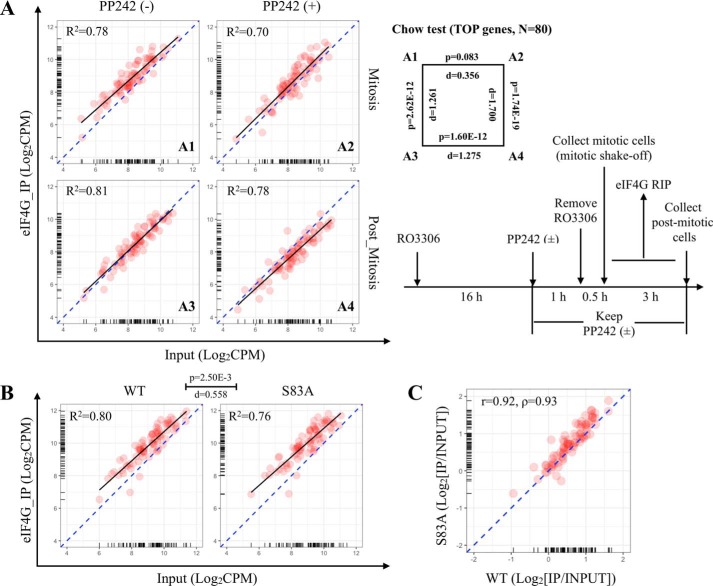
**Active mitotic 5′-TOP translation in HeLa cells.** *A*, HeLa cells were synchronized at the G_2_/M boundary with CDK1 inhibitor RO3306 treatment (10 μm; 16 h) and mTOR inhibitor PP242 treatment (5 μm; 1 h) and then released into mitosis by removing RO3306 (keeping PP242 in the medium). After incubating for 30 min, mitotic cells were collected by mitotic shake-off and lysed immediately for eIF4G RIP-Seq. The remaining cells were collected as postmitotic cells 3 h later and lysed for eIF4G RIP. The scatterplots summarize eIF4G RIP-Seq results for 5′-TOP genes. The *x axis* and *y axis* represent the abundance of transcripts in the input and eIF4G immunoprecipitated (*IP*) RNA, respectively. log_2_ cpm indicates log-transformed counts per million reads. The *black line* is the regression line for 5′-TOP gene dots (*n* = 80) based on the linear model. *R*^2^ indicates the fitness of the linear model. *p* and *d* (effect size based on *F* value) values for different comparisons (*right*) are calculated by Chow test (the null hypothesis asserts no difference in coefficients of linear models). *B*, eIF4G RIP-Seq was performed on mitotic shake-off–collected WT 4E-BP1 or 4E-BP1^S83A^ mutant HeLa cells. Results for 5′-TOP genes are presented. *C*, -fold change (IP/input) values of 5′-TOP gene transcripts between WT 4E-BP1 and 4E-BP1^S83A^ mutant HeLa cells are highly correlated. *r* indicates the Pearson correlation coefficient, and ρ indicates the Spearman rank correlation coefficient. The averaged result for three independent biological experiments is presented.

To investigate mitotic translation of individual gene transcripts, RNA binding to the translation initiation complex eIF4F was examined by eIF4G RIP-Seq. As shown in Fig. 7*A*, HeLa cells were arrested at the G_2_/M boundary with RO3306, released, and synchronized for mitotic entry. Mitotic cells were collected by shake-off, whereas attached cells were allowed to progress into postmitosis. Harvested cell pellets were then subjected to RNA-Seq and eIF4G RIP-Seq. The results for 5′-TOP genes (44) are shown in Fig. 7. Total transcriptome RIP-Seq analyses are shown in Fig. S6.

Most 5′-TOP gene transcripts were abundantly expressed in cells and proportionally bound to eIF4G during mitosis and postmitosis (linear least-squares fit, *R*^2^ = 0.70–0.81) (Fig. 7*A*). This expression-translation profile for 5′-TOP transcripts was not significantly changed by PP242 treatment in mitosis-enriched cells (Chow test, *p* = 0.083; effect size, *d* = 0.356), which is consistent with mTOR independence. Postmitotic eIF4G binding of 5′-TOP transcripts was significantly reduced compared with mitotic 5′-TOP eIF4G binding (Chow test, *p* = 2.6E−12; effect size, *d* = 1.261). Postmitotic cells treated with PP242 had a further decrease in eIF4G engagement compared with untreated postmitotic cells (Chow test, *p* = 1.60E−12; effect size, *d* = 1.275), consistent with mTOR-dependent translation of 5′-TOP transcripts during interphase.

A similar analysis was performed on mitosis-enriched 4E-BP1^WT^ and 4E-BP1^S83A^ cells, shown in Fig. 7*B*. Although 5′-TOP transcript eIF4G engagement was marginally changed in 4E-BP1^S83A^ HeLa cells compared with 4E-BP1^WT^ HeLa cells (Chow test, *p* = 2.50E−3; effect size, *d* = 0.558), this difference was lost when RIP-Seq variance (biological repeats) for both populations was taken into consideration (Fig. S7*A*). Furthermore, no clear pattern of 5′-TOP transcript translation change was evident (Pearson correlation, *r* = 0.92; Spearman rank correlation, ρ = 0.93) (Fig. 7*C*). Also, a similar analysis of total transcript translation (Fig. S7*B*) did not confidently identify an RNA population affected by S83A substitution.

## Discussion

Our study was performed to catalog mitotic and interphase 4E-BP1 phospho-isoforms and to assess their interactions with the translation initiation protein eIF4E. This was examined by eIF4E co-IP followed by 2D-gel electrophoresis and by 4E-BP1:eIF4E PLA. We found heterogeneous 4E-BP1 phosphorylations within both mitotic and interphase cells. The majority of mitotic 4E-BP1 isoforms are hyperphosphorylated at four or more sites (δ-4E-BP1) and do not bind eIF4E. A fraction of mitotic phosphorylated 4E-BP1 lacking Thr-37/Thr-46 phosphorylation retained their ability to interact with eIF4E, which has been overlooked in previous studies (34, 35, 39, 40).

There are several important caveats that should be considered when interpreting our findings. 1) STLC-induced mitotic arrest was used in our study and is anticipated to inhibit protein synthesis as with nocodazole. This method achieves high rates (>60%) of mitotic arrest for HeLa cells but will still have substantial contamination of interphase cells (Fig. S2*A*), which complicates the analysis. For example, phospho-isoforms labeled B1, C1, and D1 disappear with mTOR inhibition in STLC-treated cells, but we cannot distinguish whether these isoforms represent true mitotic phospho-isoforms or contaminating interphase phospho-isoforms (Fig. 3). About 5% of untreated, asynchronous HeLa cells undergo mitosis at any given time, and so mitotic contamination of asynchronous cells is less of a concern. Furthermore, STLC treatment, like nocodazole treatment, may nonspecifically inhibit translation. This effect, if present, is downstream of 4E-BP1 phosphorylation, and we see similar 4E-BP1 phosphorylation patterns for STLC-arrested cells compared with mitotic cells isolated by shake-off without pharmacologic mitotic arrestors (Fig. S2*B*) 2) Commercial p-4E-BP1^S65^ antibody has specific reactivity to the p-4E-BP1^S65^ epitope but cross-reacts with human p-4E-BP1^S101^, depending on the dilution of the antibody and the amount of p-4E-BP1^S101^ epitope (15). The positive p-4E-BP1^S65^ signal for EB isoforms of 4E-BP1 might represent Ser-101 phosphorylation of 4E-BP1 because Ser-65 phosphorylation has been previously described only in hyperphosphorylated isoforms of 4E-BP1 that have no interaction with eIF4E (19, 20), consistent with the weak or undetectable PLA signals between eIF4E and p-4E-BP1^S65/S101^ (Fig. 5*A*) 3) The two priming threonine sites, Thr-37 and Thr-46, have identical epitope sequences, and the available commercial p-4E-BP1^T37/T46^ antibody cannot distinguish between single Thr-37 or Thr-46 phosphorylation or between combined Thr-37/Thr-46 phosphorylations. Also, priming-site phosphorylation does not change the electrophoretic mobility of 4E-BP1 on one-dimensional SDS-PAGE (19, 45) 4) Isoelectric focusing resolves protein by charge; some of the species (“dots”) observed on 2D gel may well be composed of a mixture of species with similar charge but are actually phosphorylated at different sites.

Cap-dependent translation during mitosis is technically difficult to measure because mitosis is short (<1.5 h) as well as rare in cultured cells (∼5%), and spindle assembly inhibitors nonspecifically inhibit protein synthesis, possibly through activated downstream phosphorylation of eIF2α (35, 40). There is, however, substantial evidence from multiple studies that cap-dependent translation is active during mitosis (35, 39, 43), suggesting that the accepted dogma for a shift from cap-dependent to cap-independent translation during mitosis should be revisited. We found that eIF4G:eIF4E interaction was not inhibited during mitosis but was slightly increased (Figs. 5*B*, 1*A*, and 2, *A* and *B*). Intriguingly, previous studies on eIF4G also demonstrated enhanced assembly of eIF4F complex (eIF4G:eIF4A interaction) during nocodazole-induced mitosis in which protein synthesis was inhibited (41). Consistent with these findings, eIF4G RIP-Seq in HeLa cells demonstrated that 5′-TOP gene translation initiation is still active and mTOR-independent during mitosis (Fig. 7). However, we did not find that translation of these transcripts was related to the status of 4E-BP1^S83^ phosphorylation in HeLa cells. We cannot exclude the possibility that this effect is cell line–dependent; for example, HeLa cells have reduced mitotic translation compared with BJ-T cells (35). Alternatively, 4E-BP1^S83^ phosphorylation may be related to a nontranslational signaling pathway or may be coincidental to CDK1.

It is widely accepted that the interaction between eIF4E and 4E-BP1 is regulated by the multisite phosphorylation of 4E-BP1. However, some studies have shown that Thr-37/Thr-46 phosphorylation is sufficient to dissociate 4E-BP1 from eIF4E and that Ser-65 phosphorylation is dispensable for the regulation of 4E-BP1:eIF4E interaction (16, 25, 45, 46). A recent study using far-Western blotting supported Thr-46 phosphorylation as key in controlling 4E-BP1:eIF4E interaction (37). In this study, our co-IP data as well as PLA data indicate that no single 4E-BP1 phosphorylation is sufficient to block 4E-BP1 sequestration of eIF4E *in vivo*; rather it is a combination of phosphorylations that results in the loss of eIF4E interaction with 4E-BP1. The 2D-gel data (Fig. 4) suggest that phosphorylation at both Thr-37 and Thr-46 on 4E-BP1 is the critical event in the dissociation of 4E-BP1 from eIF4E and support the notion that further Thr-70 or Ser-65 phosphorylation is dispensable in controlling 4E-BP1:eIF4E interaction (46). This differs from the canonical two-step model (19). Usually, the eIF4E-bound 4E-BP1 migrated into two or more bands after one-dimensional SDS-PAGE. These two bands both can be visualized using p-4E-BP1^T37/T46^ and p-4E-BP1^T70^ antibodies, leading to the misinterpretation that only hyperphosphorylated 4E-BP1 (p-4E-BP1^T37/T46, S65, T70^) can dissociate from eIF4E. However, due to the limitation of the resolution of SDS-PAGE, the two bands actually correspond to multiple, alternative overlapping isoforms of 4E-BP1 as demonstrated by 2D-gel electrophoresis (Fig. 4). Unlike the previously proposed canonical model for the dissociation of eIF4E from 4E-BP1, wherein higher-order phosphorylations are entirely predicated upon priming phosphorylations at Thr-37 and Thr-46, these priming phosphorylations are not required for 4E-BP1 hyperphosphorylation during mitosis because CDK1/cyclin B can substitute for mTOR to phosphorylate 4E-BP1 at multiple sites (35). Previous studies have shown that Ser-2448 phosphorylation of mTORC1 is reduced during mitosis (47); however, assessment of mitotic mTOR activity is complicated. 4E-BP1 or ribosomal S6 kinase (S6K1) phosphorylations are frequently used as a surrogate readouts for mTORC1 activity, but both of these proteins are also phosphorylated by kinases other than mTORC1 during mitosis (35, 48, 49). The bulk of mitotic 4E-BP1 phosphorylation remains resistant to PP242, suggesting that kinases other than mTOR are primarily responsible for mitotic 4E-BP1 phosphorylation. We cannot conclude that mTORC1 plays no role in mitotic 4E-BP1 phosphorylation and it may act in concert with CDK1/cyclin B to generate fully phosphorylated 4E-BP1 isoforms during mitosis. It is desirable to directly determine the status of mTORC1 in mitosis, for example whether mTORC1 is still in a dimer active form (50, 51). Our study also confirms that p-4E-BP1^S83^ is a unique marker for mitotic cells. Ser-83 phosphorylation alone is insufficient to block 4E-BP1 sequestration of eIF4E. Interestingly, a recent study reported another CDK could phosphorylate 4E-BP1, relying on mTOR-priming phosphorylation (52), whereas CDK1 can phosphorylate 4E-BP1 at various residues independently of mTOR kinase. Taken together, our investigation of 4E-BP1:eIF4E interaction during the cell cycle reveals a complex accounting of the phosphorylation profile of 4E-BP1 isoforms bound to eIF4E.

## Experimental procedures

### Cell culture and transfection

HEK 293 and HeLa cells were maintained in Dulbecco's modified Eagle's medium (Corning Cellgro) supplemented with 10% fetal bovine serum. HEK 293 and HeLa cells were transfected with eIF4E expression plasmids using polyethylenimine (Sigma-Aldrich) and reseeded 12–16 h post-transfection to avoid confluence. Transfected cells were harvested 48 h post-transfection.

### Plasmids and antibodies

HA-tagged and FLAG-tagged eIF4E expression plasmids were constructed by cloning eIF4E to AfeI and SbfI sites on the pLVX-EF-puro plasmid (53). pLVX-EF-4E-BP1^WT^ and pLVX-EF-4E-BP1^S83A^ expression plasmids were generated based on previous constructs (34). Doxycycline-inducible 4E-BP1^T37A^ and 4E-BP1^T46A^ plasmids were constructed by cloning corresponding 4E-BP1 mutant fragments to AfeI and SbfI sites on the pLenti-TRE-MCS-EF-Puro-2A-rTet plasmid (54). DNA constructs used in this study are listed in Table S1.

The following primary antibodies were used in this study: total 4E-BP1 (53H11, Cell Signaling Technology), phospho-4E-BP1^T37/T46^ (236B4, Cell Signaling Technology), phospho-4E-BP1^T70^ (9455, Cell Signaling Technology), phospho-4E-BP1^S65^ (9451, Cell Signaling Technology), phospho-4E-BP1^S83^ (ABE2889, Millipore), eIF4E (C46H6, Cell Signaling Technology), eIF4GI (D6A6, Cell Signaling Technology), eIF4E (A-10, Santa Cruz Biotechnology), eEF2 (2332, Cell Signaling Technology), HA tag (16B12, BioLegend), and FLAG tag (M2, Sigma-Aldrich).

### Construction of 4E-BP1–knockout and mutant cell lines

HeLa 4E-BP1–knockout cell line was established using the CRISPR/Cas9 strategy (55) (target sequence, 5′-TGAAGAGTCACAGTTTGAG-3′). The established cell line was verified by sequencing and immunoblotting. To construct 4E-BP1 mutant stable cell lines, 4E-BP1 WT and its S83A mutant were re-expressed in the HeLa 4E-BP1-knockout cell line through lentiviral transduction.

### Cell cycle synchronization

Mitotic cells were enriched by STLC treatment (5 μm; 16 h) (56) or by mitotic shake-off. For the latter, cells were treated with 10 μm CDK1 inhibitor RO3306 for 16 h to arrest cells at the G_2_/M boundary, and then the cells were released into mitosis by removing RO3306. After 30 min, mitotic cells were collected by mechanical shake-off.

### Immunoprecipitation and immunoblotting

Cells were lysed in nondenaturing RIPA buffer (50 mm Tris·HCl (pH 7.4), 150 mm NaCl, 0.5% Triton X-100, 0.5% sodium deoxycholate, 2 mm Na_3_VO_4_, 2 mm NaF) supplemented with protease inhibitors (Roche Applied Science). Lysates were incubated with protein A/G–Sepharose beads (Santa Cruz Biotechnology) and anti-FLAG or anti-HA tag antibodies overnight at 4 °C. Beads were collected, washed four times with RIPA buffer, and boiled in SDS loading buffer. Samples were subjected to 12% SDS-PAGE and transferred to nitrocellulose membranes. Membranes were blocked with 5% milk and incubated with primary antibodies overnight at 4 °C. After washing, blots were subsequently incubated with IRDye-labeled anti-rabbit or anti-mouse secondary antibodies (LI-COR Biosciences) and analyzed by Odyssey IR scanning (LI-COR Biosciences).

### m^7^GTP cap-binding assay

Cells were lysed in nondenaturing RIPA buffer supplemented with protease inhibitors (Roche Applied Science). Lysates were incubated with 30 μl of m^7^GTP-Sepharose beads (Jena Bioscience) overnight at 4 °C. Beads were collected, washed four times with RIPA buffer, and boiled in 1× SDS loading buffer. Samples were subjected to 12% SDS-PAGE and immunoblotting.

### PLA

Cells grown on coverslips (Thomas Scientific) were fixed with 4% paraformaldehyde for 30 min followed by permeabilization with 0.2% Triton X-100 for 10 min. PLA was performed by using the Duolink PLA kit (Sigma-Aldrich). After permeabilization, samples were treated with blocking solution for 60 min at 37 °C inside a humidity chamber. Then samples were incubated with primary antibodies overnight at 4 °C inside a humidity chamber. The following antibodies were used in PLA: eIF4E (A-10; 1:400), phospho-4E-BP1^S83^ (ABE2889; 1:400), total 4E-BP1 (53H11; 1:1000), phospho-4E-BP1^T37/T46^ (236B4; 1:1000), phospho-4E-BP1^T70^ (9455; 1:120), phospho-4E-BP1^S65^ (9451; 1:1000), eIF4GI (D6A6; 1:600), and eEF2 (2332; 1:600). Samples were incubated with PLA secondary antibodies (1:10) inside a humidity chamber for 60 min at 37 °C. Detection steps, including ligation, amplification, and DAPI staining, were carried out according to the manufacturer's instructions. Images were captured using fluorescence and confocal microscopy (Olympus). Images were processed with ImageJ.

### 2D-gel electrophoresis

Cells were lysed in RIPA buffer supplemented with protease inhibitors (Roche Applied Science) with a final lysate protein concentration above 10 μg/μl. Cleared lysates (400–500 μg) were diluted with rehydration buffer (Bio-Rad) to 220 μl and then loaded to immobilized pH 3–6 gradient strips (Bio-Rad) for rehydration overnight. The rehydrated strips were focused with linear voltage ramping for 2 h at 200 V, 2 h at 500 V, and 16 h at 800 V. After focusing, the balanced strips were subjected to SDS-PAGE for second-dimensional separation and immunoblotting.

For immunoprecipitated samples, the final collected beads were boiled with 20 μl of 2% SDS (57) and centrifuged to collect the supernatants (cooled to room temperature). The samples were then diluted with rehydration buffer to 220 μl prior to two-dimensional gel electrophoresis as described above.

### Click-iT labeling assay

Cells were cultured in 6-well plates with or without drug treatment. For labeling newly synthesized proteins, cells were washed with methionine-depleted medium once and cultured with methionine-depleted medium. After incubating for 15 min, cells were treated with HPG (50 μm) for 30 min. Cycloheximide (100 μg/ml) was added concurrently to block new protein synthesis. Cells were collected and fixed with 4% paraformaldehyde for 30 min followed by permeabilization with 0.2% Triton X-100 for 10 min. Incorporated HPG was labeled with Alexa Fluor 488 azide using Click-iT HPG kits (Life Technologies). Cells were stained with p-H3^S10^ antibody (3458, Cell Signaling Technology) to label the mitotic cell population. HPG incorporation in cells was analyzed by flow cytometry.

### RIP-Seq

eIF4G RIP were performed using the RIP-Assay kit (RN1001, MBL International). Collected cell pellets were lysed in 800 μl of kit-provided lysis buffer supplemented with protease inhibitors (Roche Applied Science), RiboLock RNase inhibitor (Thermo Fisher), and dithiothreitol (DTT) on ice for 10 min. Lysed samples were centrifuged at 12,000 × *g* for 5 min at 4 °C to collect the supernatant (cell lysate). 80 μl of supernatant was set aside as input, and the remaining supernatant was divided into two groups. Lysates were incubated with 30 μl of protein A/G–Sepharose beads (Santa Cruz Biotechnology) and 5 μl of kit-provided rabbit IgG or eIF4G antibody (RN002P, MBL International) overnight at 4 °C. Beads were collected and washed three times with kit-provided wash buffer supplemented with DTT. The immunoprecipitated and input RNA was extracted using TRIzol (Thermo Fisher). Double-strand cDNA libraries were prepared with a SMART-seq Ultra Low Input kit (Takara Clontech). Double-strand cDNA libraries were fragmented and indexed using a Nextera XT DNA library preparation kit (Illumina). The quality of extracted RNA, double-strand cDNA libraries, and Nextera XT DNA libraries was determined on a Bioanalyzer2100 (Agilent). Illumina NextSeq 500 sequencing was performed in paired-end read mode with 75 cycles.

Reads were trimmed and filtered to remove adaptor sequences with Trim Galore and Cutadapt programs. Trimmed sequences were aligned to human genome (hg19) with CLC Genomics Workbench (Qiagen). Data were analyzed using CLC Genomics Workbench and R. The 5′-TOP gene list was adapted from a previous study (44). The sequencing data reported in this paper have been deposited in the Gene Expression Omnibus database under accession number GSE131668.

## Author contributions

R. S., Y. C., and P. M. conceptualization; R. S. data curation; R. S., Y. C., and P. S. M. formal analysis; R. S. investigation; R. S. writing-original draft; E. C. and C. V. resources; C. V., Y. C., and P. S. M. writing-review and editing; Y. C. and P. S. M. supervision; Y. C. and P. S. M. funding acquisition; E. C. and C. V. established and prepared 4E-BP1 plasmid constructs, 4E-BP1–knockout cell lines, and phospho-4E-BP1 Ser-83 antibody.

## Supplementary Material

Supporting Information

## Footnotes

5 The abbreviations used are:

4E-BP1: eIF4E-binding protein 1
eIF: eukaryotic translation initiation factor
CDK: cyclin-dependent kinase
RIP-Seq: RNA immunoprecipitation sequencing
mTORC1: mechanistic target of rapamycin complex 1
TOP: terminal oligopyrimidine
PLA: proximity ligation assay
STLC: *S*-trityl-l-cysteine
EB: eIF4E-binding
p-4E-BP1: phosphorylated 4E-BP1
co-IP: coimmunoprecipitation
HPG: l-homopropargylglycine
HEK: human embryonic kidney
HA: hemagglutinin
RIPA: radioimmune precipitation assay
DAPI: 4′,6-diamidino-2-phenylindole.
