# Supplementary material for: Mitosis-related phosphorylation of the eukaryotic translation suppressor 4E-BP1 and its interaction with eukaryotic translation initiation factor 4E (eIF4E)
Source: J Biol Chem. 2019 Jun 14;294(31):11840–52. doi: 10.1074/jbc.RA119.008512 (PMC6682726; doi:10.1074/jbc.RA119.008512)
Supplement: Supporting Information [file supp_294_31_11840__index.html]

Mitosis-related phosphorylation of the eukaryotic translation suppressor 4E-BP1 and its interaction with eukaryotic translation initiation factor 4E (eIF4E) — Mitotic 4E-BP1:eIF4E interaction — Mitosis-related phosphorylation of the eukaryotic translation suppressor 4E-BP1 and its interaction with eukaryotic translation initiation factor 4E (eIF4E) — Mitotic 4E-BP1:eIF4E interaction — Supporting Information 

# Mitosis-related phosphorylation of the eukaryotic translation suppressor 4E-BP1 and its interaction with eukaryotic translation initiation factor 4E (eIF4E)

## Supporting Information

- Supporting Information - Fig. S1-S7 and Table S1
