# Supplementary material for: Mitosis-related phosphorylation of the eukaryotic translation suppressor 4E-BP1 and its interaction with eukaryotic translation initiation factor 4E (eIF4E)
Source: J Biol Chem. 2019 Jun 14;294(31):11840–52. doi: 10.1074/jbc.RA119.008512 (PMC6682726; doi:10.1074/jbc.RA119.008512)
Supplement: Supporting Information [file supp_RA119.008512_144406_3_supp_345539_psw1kp.pdf]

Mitosis-related 4E-BP1 phosphorylation and eIF4E interaction

Rui Sun[1, 3], Erdong Cheng[1, 3#], Celestino Velásquez[1, 3†], Yuan Chang[2, 3]* and Patrick S. Moore[1, 3]*

From [1]Department of Microbiology and Molecular Genetics, University of Pittsburgh, Pittsburgh, PA 15213; [2]Department of Pathology, University of Pittsburgh, Pittsburgh, PA 15213; [3]Cancer Virology Program, UPMC Hillman Cancer Center, Pittsburgh, PA 15213

[#]Present address: Department of Urology, University of Pittsburgh, Pittsburgh, PA 15232
[†]Present address: Department of Biology and Chemistry, Oral Roberts University, Tulsa, OK 74171

*To whom correspondence should be addressed: Yuan Chang (yc70@pitt.edu) or Patrick S. Moore (psm9@pitt.edu), Cancer Virology Program, UPMC Hillman Cancer Center, Pittsburgh, PA 15213; Tel. (412) 623-7721.

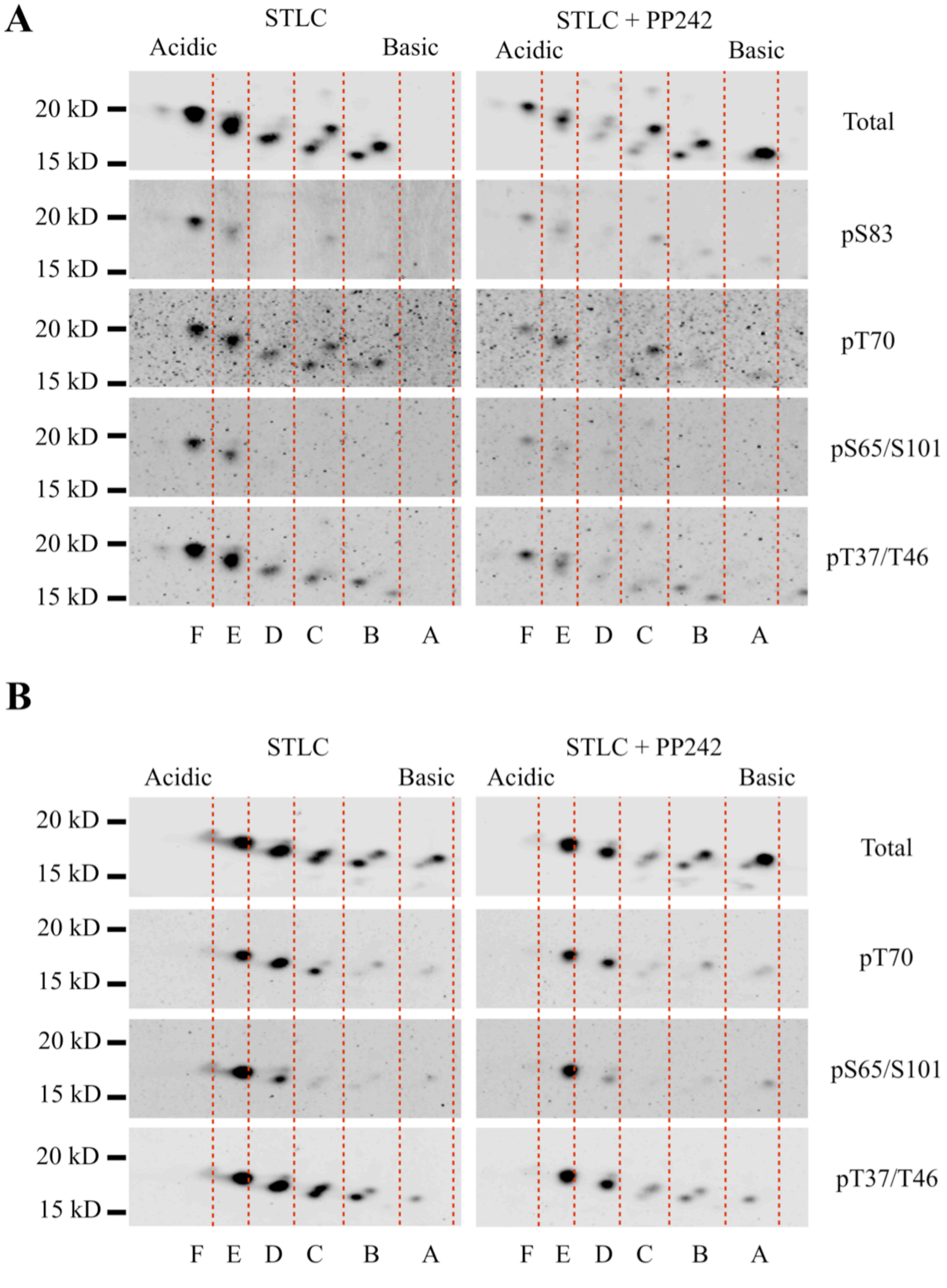

Figure S1. **(A)** Wild-type 4E-BP1 or **(B)** 4E-BP1^{S83A} mutant was stably expressed in HeLa-4E-BP1-knockout cells. Cell lysates were collected from STLC-arrested (5 μM, 16 h) cells treated with or without mTOR inhibitor PP242 (5 μM, 4 h). Cell lysates were then subjected to 2D-gel electrophoresis (isoelectric focusing at pH 3-6), followed by immunoblotting with different phospho-specific and total 4E-BP1 antibodies.

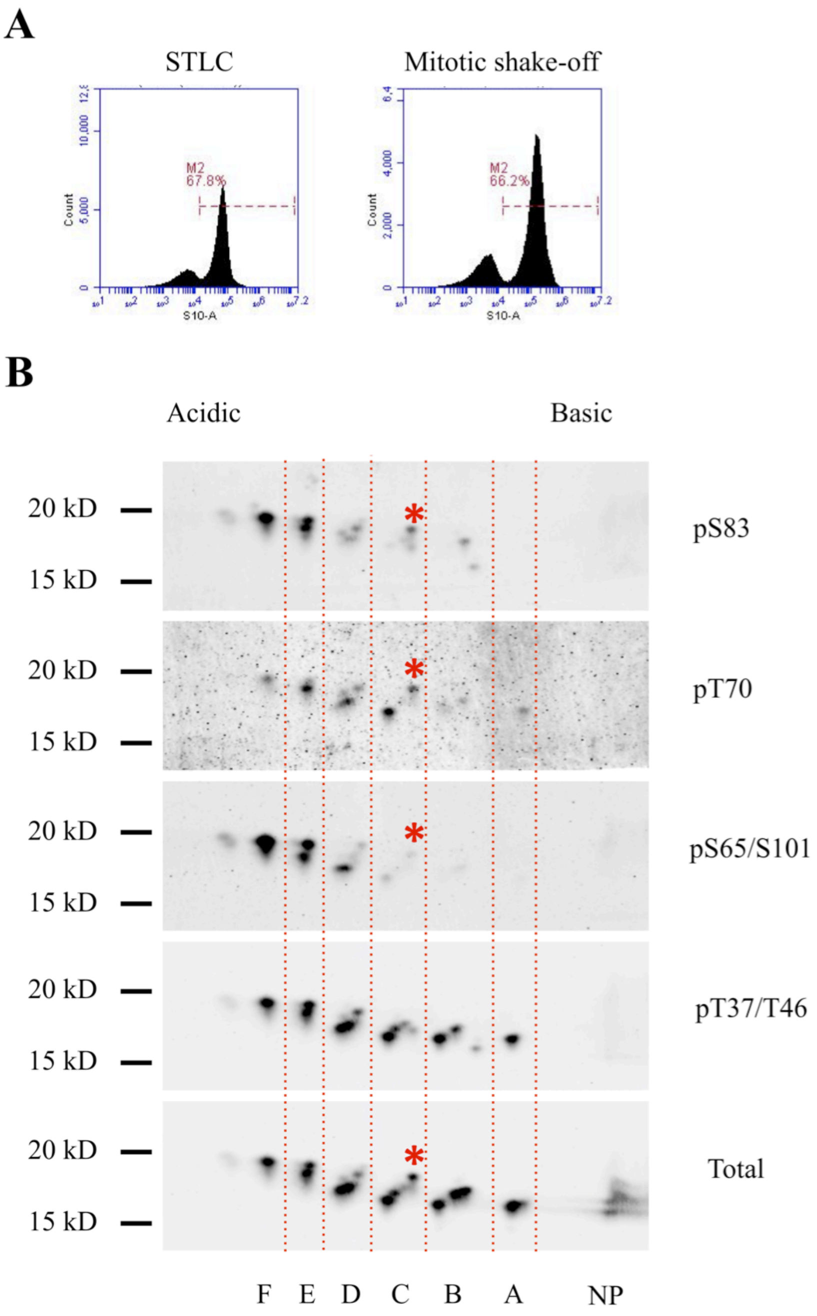

Figure S2. HeLa cells were synchronized at the G2/M boundary with CDK1 inhibitor RO3306 treatment (10 µM, 16 h) and then released into mitosis by removing RO3306. Mitotic cells were collected by mechanical shake-off. **(A)** Cell cycle analysis on cells collected by mechanical shake-off and STLC treatment. Cells were fixed and stained with phospho-histone $H3^{S10}$ antibody. Mitotic ratio was determined by positive phospho-histone $H3^{S10}$ staining. **(B)** Cell lysates were subjected to 2D-gel electrophoresis (isoelectric focusing at pH 3-6), followed by immunoblotting with phospho-specific and total 4E-BP1 antibodies. The 4E-BP1 EB-**γ** isoform is indicated by *.

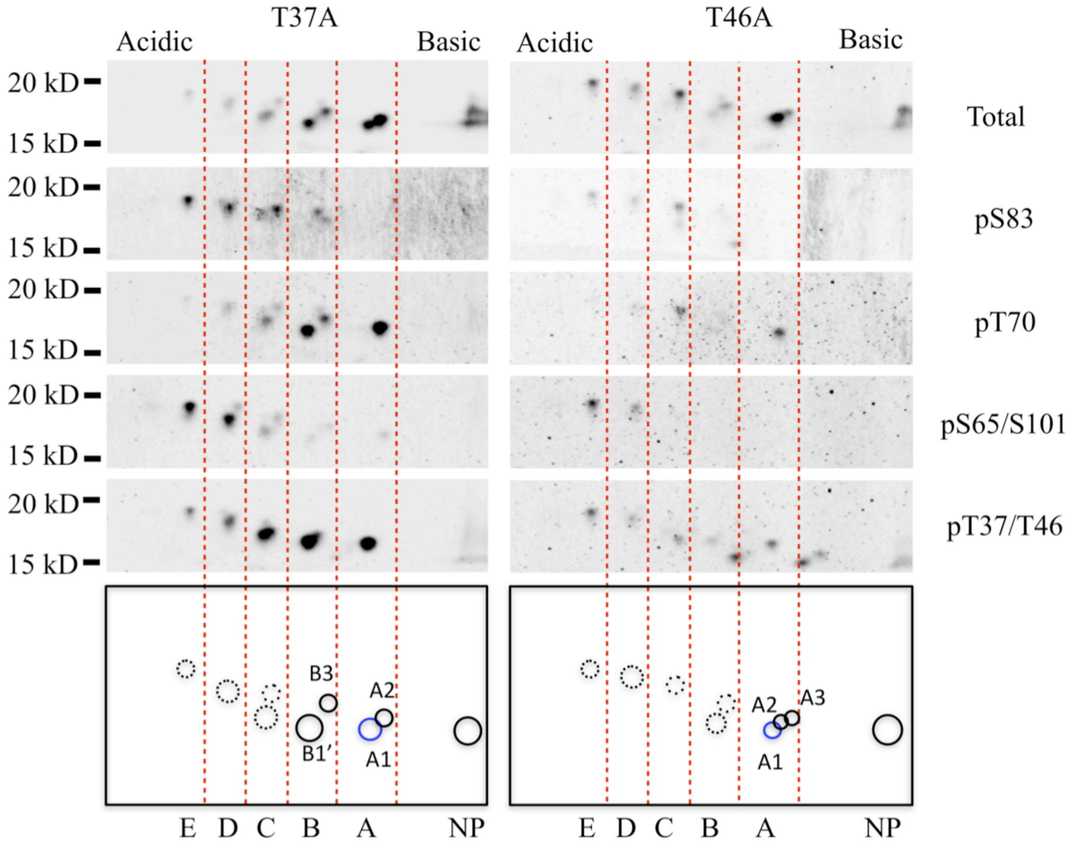

Figure S3. 4E-BP1^{T37A} or 4E-BP1^{T46A} mutants were stably expressed in HeLa-4E-BP1-knockout cells. Cell lysates were collected and then subjected to 2D-gel electrophoresis (isoelectric focusing at pH 3-6), followed by immunoblotting with different phospho-specific and total 4E-BP1 antibodies. Dot B1', positive for T37/T46 and T70 phosphorylation, was aberrantly accumulated in 4E-BP1^{T37A} mutant cells. The original dot B1, positive for T37/T46 phosphorylation, was absent in either 4E-BP1^{T37A} or 4E-BP1^{T46A} mutant cells.

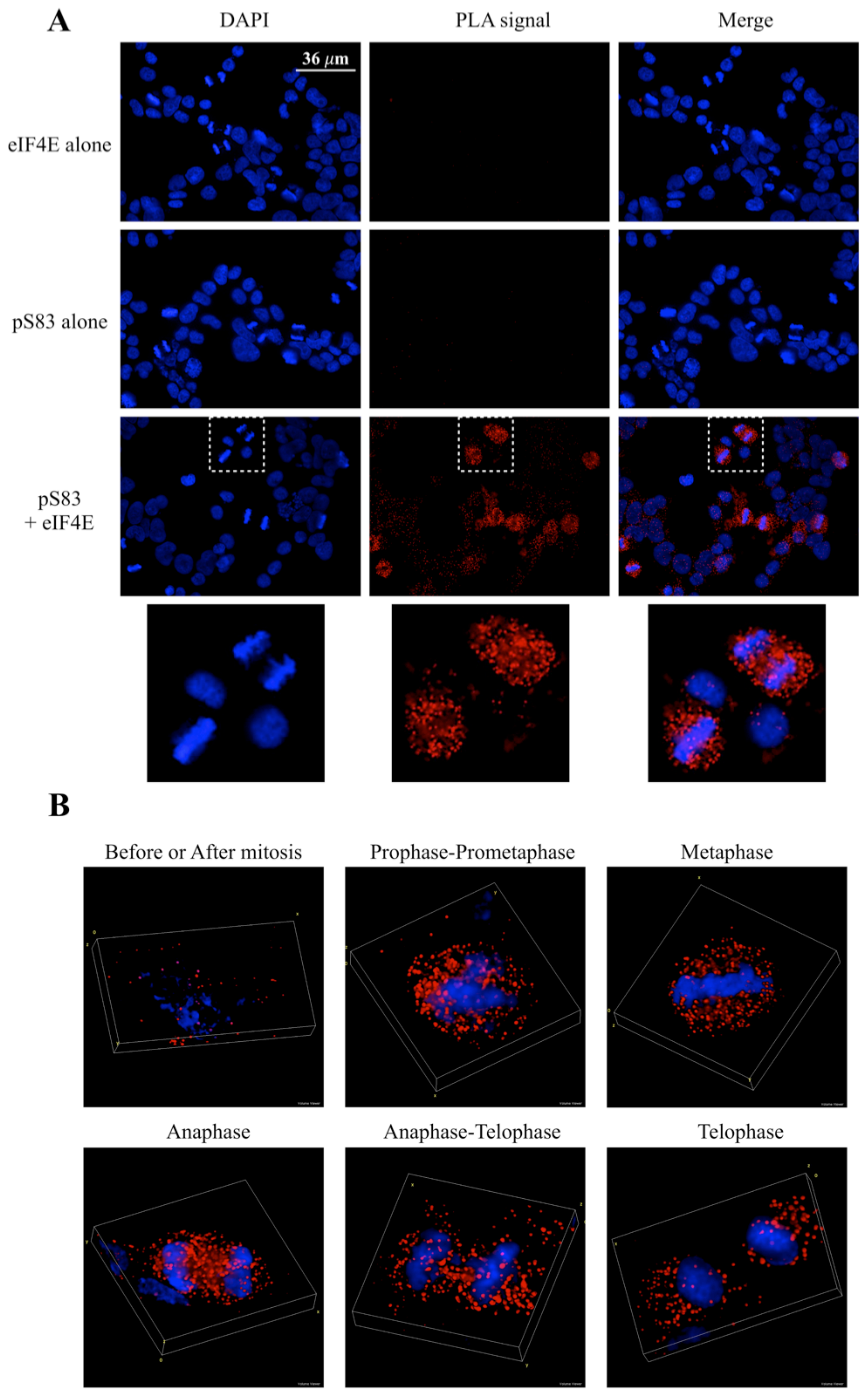

Figure S4. HeLa cells were synchronized at the G2/M boundary with CDK1 inhibitor RO3306 treatment (10 μM, 16 h) and then released into mitosis by removing RO3306. After 60 min, cells were fixed and permeabilized. **(A)** Proximity ligation assays (PLA) were performed using mouse eIF4E and/or rabbit p-4E-BP1^{S83} antibodies. Cell nuclei were stained with DAPI (blue). PLA signals obtained from rolling circle amplification are designated by red fluorescence. Images were captured by fluorescence microscopy (40X). **(B)** Samples in (A) were further examined using confocal microscope with Z-stack scanning (100X). 3D-images were reconstructed with Image J.

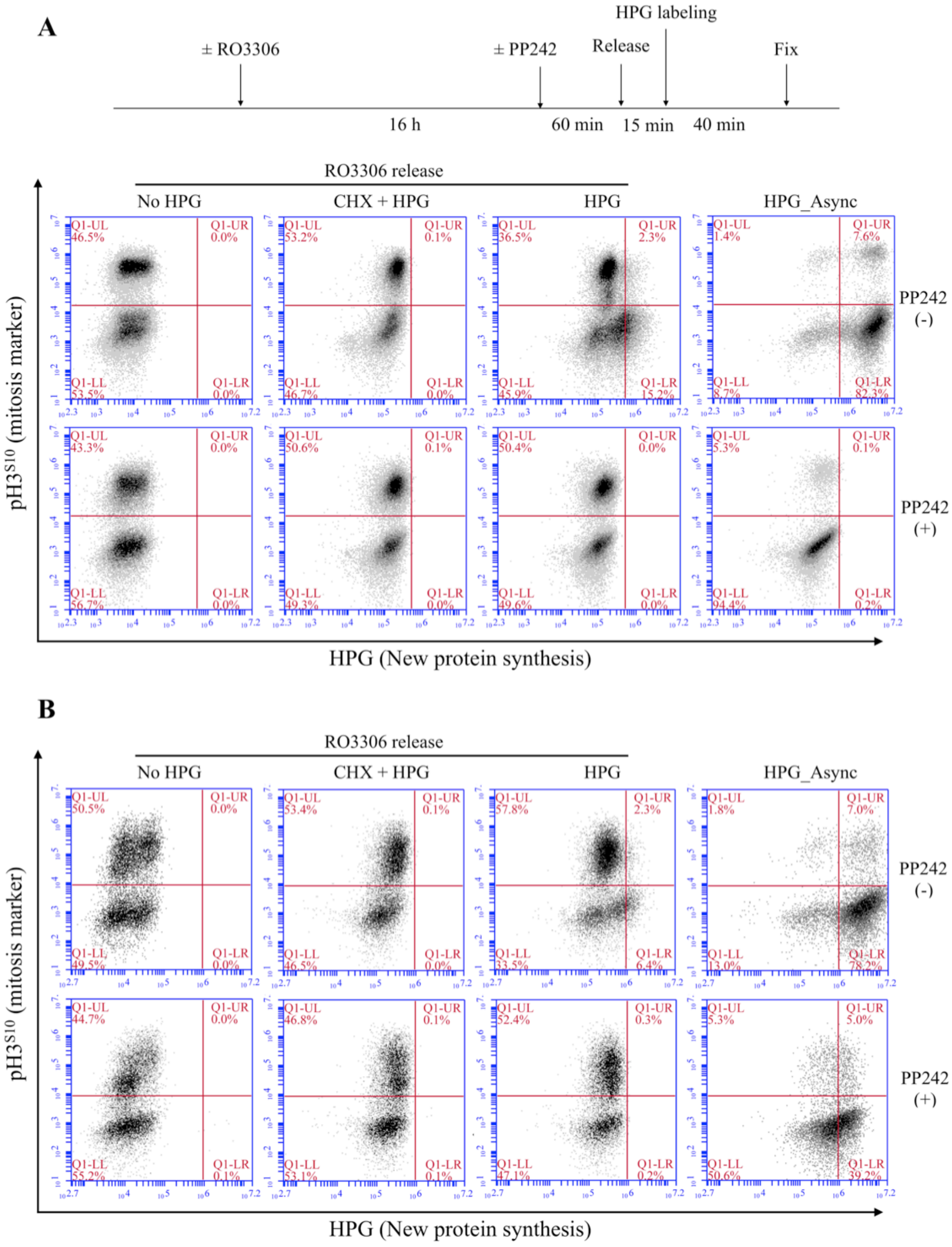

Figure S5. Native HeLa **(A)** or HeLa-4E-BP1 knockout cells **(B)** were synchronized at the G2/M boundary with CDK1 inhibitor RO3306 treatment (10 µM, 16 h) and mTOR inhibitor PP242 treatment (5 µM, 1 h), and then released into mitosis by removing RO3306 (keeping PP242 in the medium). Asynchronous cells were included as positive control. After incubating with methionine-depleted medium for 15 min, cells were treated with L-homopropargylglycine (HPG; 50 µM) for 30 min. Cycloheximide (CHX; 100 µg/mL) was added at the same time to block new protein synthesis, and used as negative control. Cells were collected and fixed for subsequent staining and flow cytometry analysis of HPG incorporation (new protein synthesis). Cells were labeled with the Alexa Fluor 488 azide by using the Click-iT HPG kits (Life Technologies) and stained with p-H3^{S10} antibody to label the mitotic cell population.

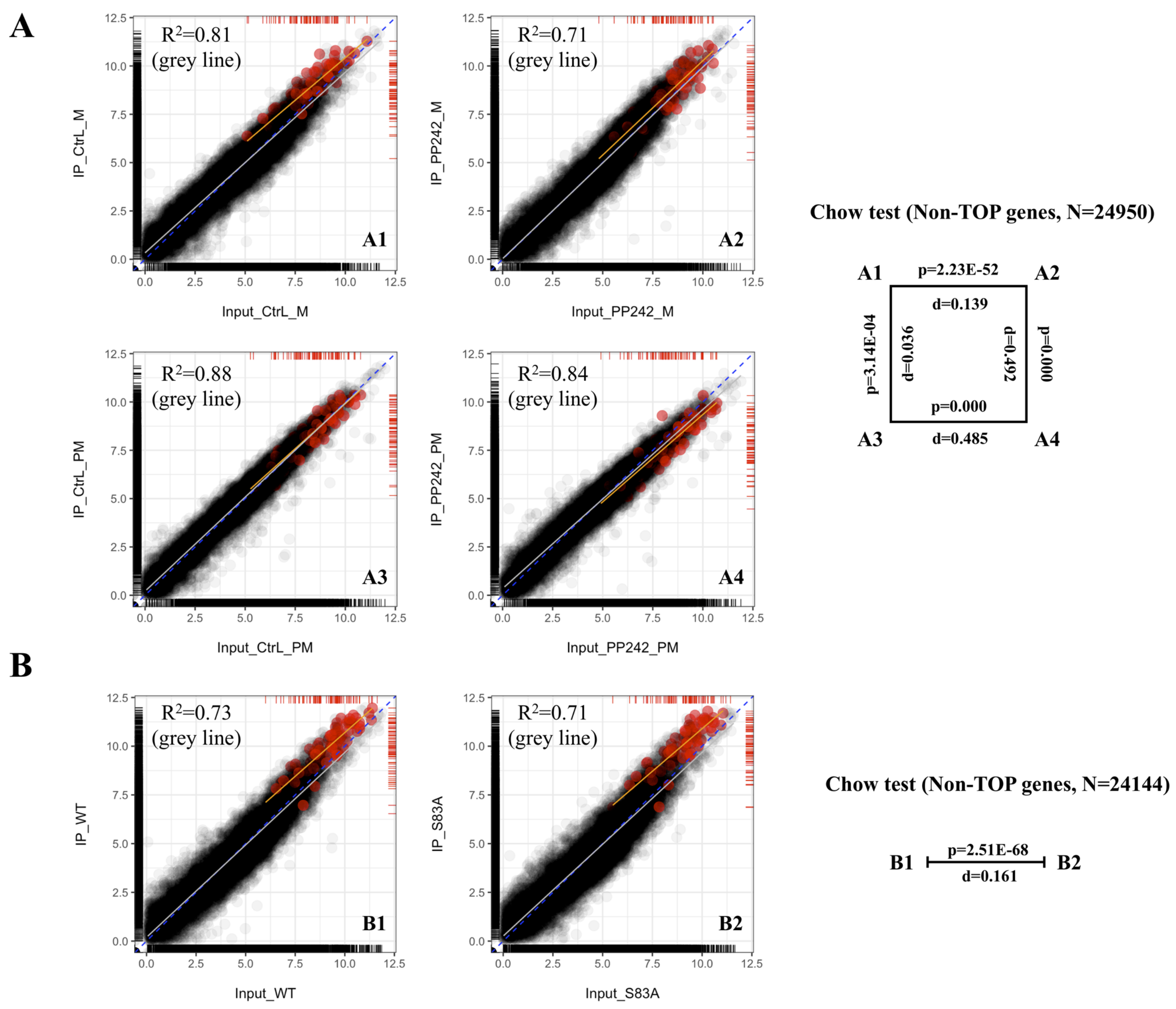

Figure S6. (A) HeLa cells were synchronized at the G2/M boundary with CDK1 inhibitor RO3306 treatment (10 µM, 16 h) and mTOR inhibitor PP242 treatment (5 µM, 1 h), and then released into mitosis by removing RO3306 (keeping PP242 in the medium). After incubating for 30 min, mitotic cells were collected by mitotic shake-off and immediately lysed for eIF4G RIP (immunoprecipitation RNA-seq). The remained cells were collected as post-mitosis cells 3 h later and lysed for eIF4G RIP. Scatterplots summarized eIF4G RIP-seq results. The x axis and y axis represent the abundance of transcripts in the Input and eIF4G immunoprecipitated (IP) RNA respectively. Log2CPM indicates Log-transformed CPM (counts per million reads). The orange line is the regression line for 5'-TOP gene dots (N=80) based on the linear model. The grey line is the regression line for non-TOP gene dots based on the linear model. R^2 indicates the fitness of the linear model. P and d (effect size based on F value) values for different comparisons (right) are calculated by Chow test (null hypothesis asserts no difference in coefficients of linear models). **(B)** eIF4G RIP-seq was performed on mitotic shake-off collected wild-type 4E-BP1 or 4E-BP1^{S83A} mutant HeLa cells. 5'-TOP genes are indicated as red dots and were further analyzed in **Fig. 7B**.

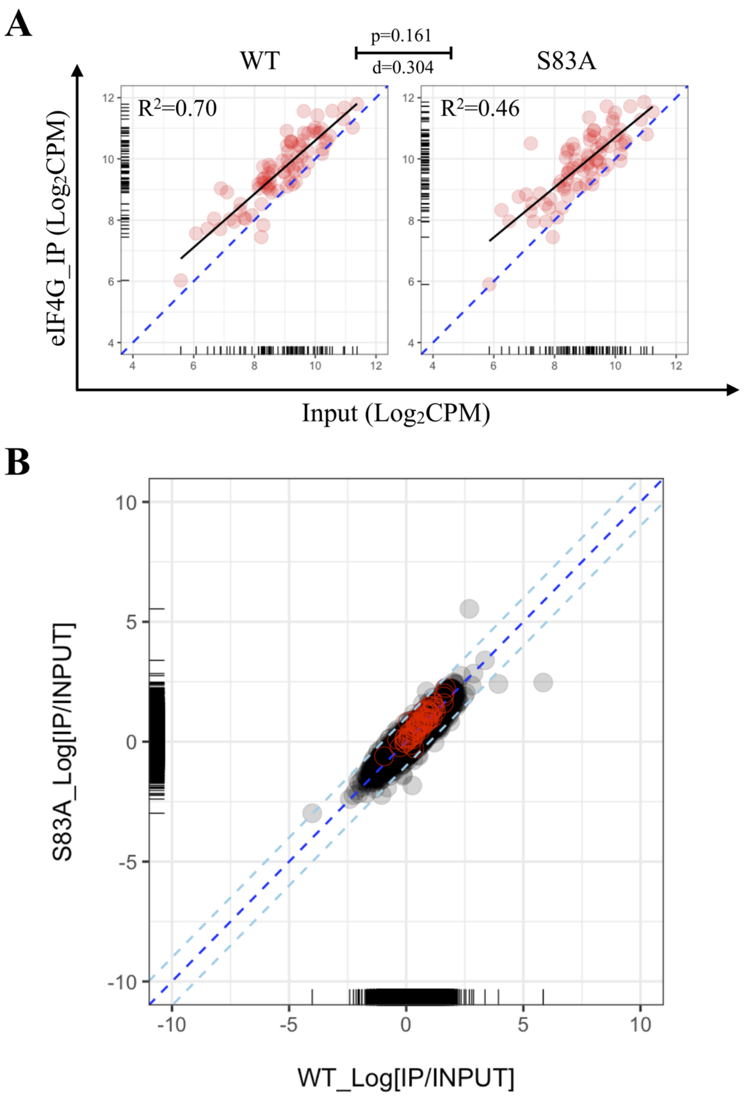

Figure S7. eIF4G RIP-seq was performed on mitotic shake-off collected wild-type 4E-BP1 or 4E-BP1^{S83A} mutant HeLa cells. **(A)** The scatterplots summarized averaged eIF4G RIP-seq results of three independent biological experiments. Results for 5'-TOP (5'-terminal oligopyrimidine) genes were presented. The x axis and y axis represent the abundance of transcripts in the Input and eIF4G immunoprecipitated (IP) RNA respectively. Log2CPM indicates Log-transformed CPM (counts per million reads). The black line is the regression line for 5'-TOP gene dots (N=80) based on the linear model. R^2 indicates the fitness of the linear model. P and d (effect size based on F value) values are calculated by Chow test (null hypothesis asserts no difference in coefficients of linear models). **(B)** Fold change (IP/Input) of total transcriptome in wild-type 4E-BP1 and 4E-BP1^{S83A} mutant HeLa cells were analyzed. Each black dot represents one gene. CPM >=10. Averaged result for three independent biological experiments is presented. Red circles indicate TOP genes.

Table S1. DNA constructs used in this study

Construct Name	CM plasmid	Note
**pLVX-EF-HA-eIF4E**	3892	HA-tagged human eIF4E
**pLVX-EF-FLAG-eIF4E**	3891	Flag-tagged human eIF4E
**pLVX-EF-4E-BP1-WT**	4346	Human 4E-BP1 wild type without tag
**pLVX-EF-4E-BP1-S83A**	4348	Human 4E-BP1 S83A mutant without tag
**pTRE-4E-BP1-T46A**	4466	Inducible human 4E-BP1 T46A mutant without tag
**pTRE-4E-BP1-T37A**	4465	Inducible human 4E-BP1 T37A mutant without tag
**pX330-sg-4E-BP1-4**	3946	To knock out 4E-BP1 by CRISPR/Cas9
